# Enhanced adsorption of phenolic compounds using biomass-derived high surface area activated carbon: Isotherms, kinetics and thermodynamics

**DOI:** 10.1007/s11356-024-32971-1

**Published:** 2024-04-05

**Authors:** Praveengouda Patil, Gautham Jeppu, Manjunath Singanodi Vallabha, Chikmagalur Raju Girish

**Affiliations:** 1 Department of Chemical Engineering, Manipal Institute of Technology, Manipal Academy of Higher Education, Manipal-576104, Karnataka India; 2 Department of Civil Engineering, BMS College of Engineering, Bengaluru-560019, India

**Keywords:** Activated carbon, Adsorption, Agricultural Biomass, *Cassia fistula*, Phenolic pollutants

## Abstract

The progress of industrial and agricultural pursuits, along with the release of inadequately treated effluents especially phenolic pollutant, has amplified the pollution load on environment. These organic compounds pose considerable challenges in both drinking water and wastewater systems, given their toxicity, demanding high oxygen and limited biodegradability. Thus, developing an eco-friendly, low-cost and highly efficient adsorbent to treat the organic pollutants has become an important task. The present investigation highlights development of a novel adsorbent (CFPAC) by activation of *Cassia fistula* pod shell for the purpose of removing phenol and 2,4-dichlorophnenol (2,4-DCP). The significant operational factors (dosage, pH, concentration, temperature, speed) were also investigated. The factors such as pH = 2 and *T* = 20°C were found to be significant at 1.6 g/L and 0.6 g/L dosage for phenol and 2,4-DCP respectively. Batch experiments were further conducted to study isotherms, kinetic and thermodynamics studies for the removal of phenol and 2,4-DCP. The activated carbon was characterised as mesoporous (specific surface area 1146 m^2^/g, pore volume = 0.8628 cc/g), amorphous and pH_PZC_ = 6.4. At optimum conditions, the maximum sorption capacity for phenol and 2,4-DCP were 183.79 mg/g and 374.4 mg/g respectively. The adsorption isotherm was better conformed to Redlich Peterson isotherm (phenol) and Langmuir isotherm (2,4-DCP). The kinetic study obeyed pseudo-second-order type behaviour for both the pollutants with *R*^2^ > 0.999. The thermodynamic studies and the value of isosteric heat of adsorption for both the pollutants suggested that the adsorption reaction was dominated by physical adsorption (Δ*H*_x_ < 80 kJ/mol). Further, the whole process was feasible, exothermic and spontaneous in nature. The overall studies suggested that the activated carbon synthesised from *Cassia fistula* pods can be a promising adsorbent for phenolic compounds.

**Graphical Abstract:**

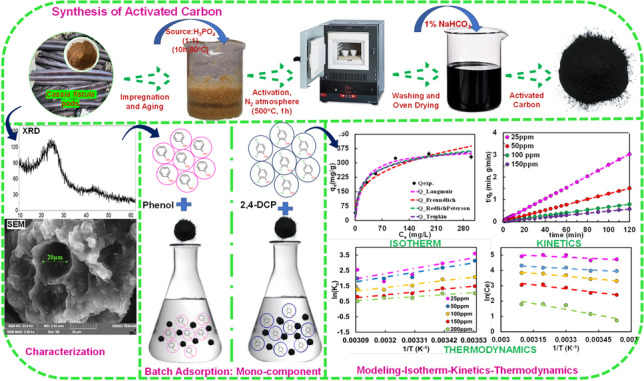

**Supplementary Information:**

The online version contains supplementary material available at 10.1007/s11356-024-32971-1.

## Introduction

With increase in the population, there is drastic rise in industrialisation and other agricultural developments from the past decade. Due to such drastic growth in population, the nation can expect a soaring demand for clean potable water. Further, an investigation by Li et al. have reported, that there will be a great dearth of water free from pollutants (J. Li et al. 2022). The reason being improper management of toxic pollutants by the industries (Al Manmi et al. 2019). Phenolic compounds which are few of the utmost used chemicals in the industries are available naturally in the environment and produced due to anthropogenic interests. The key synthetic sources of these pollutants are containing methylated and chlorinated phenols in the aquatic environment which are from industrial emissions (Nazal et al. 2021). Some of the notable key industries are coal conversion, petrochemicals and petroleum products, pharmaceuticals, paper and pulp, plastic and pesticide, wood protective chemicals, rubber-proofing, iron and steel (Adegoke and Adekola 2010; Beker et al. 2010; Boontham et al. 2020; Lorenc-Grabowska et al. 2016).

One common pollutant that is being discharged from most of these industries is phenol and its derivative say chlorophenols and 2,4-dichlorophenol (2,4-DCP) (Anku et al. 2017). These chemicals have great application at industrial level. However, the presence of phenolic pollutants even at lower concentration in the range of 5–10 μg/L has an ability to produce peculiar odour and can make it unfit for any domestic usage. As per the recommendations from the WHO, the permissible limit of total phenolic content permissible in potable water is 0.001 mg/L (Galdino et al. 2021), and the maximum contamination level quality imposes 0.1 mg/L of 2,4-DCP (Wu et al. 2022)*.* Further, the effluent discharge from the industries can also impact waterbodies and the food chain (Conde-Cid et al. 2020). In addition, the contamination can also affect human health of both acute and chronic nature on long-term exposure. Acute impacts could be breathing issues, weak muscles, skin irritation, eyes and mucous membranes. Chronic effects may be tremors, coma, and respiratory arrest, anorexia etc. (Xie et al. 2020). A notable number of the major health issues are observed in human beings. The details are listed in supplementary file Table S1. Owing to these facts, the agency US-EPA also has mandated to lower the concentration of phenol in wastewater to be below 1 ppm (Beker et al. 2010). For these reasons, it is also obligatory to separate such aromatic impurities from wastewater.

Further, to understand the scope of the subject, a survey was conducted using Scopus tool (Patil et al. 2023) for the keywords “phenol” AND “adsorption” AND “activated” AND “carbon.” The survey shows, approximately 3012 documents were published between 1963 and 2023 and the numbers are increasing. Further, it was found that the intended research is predominantly being carried out in various domains such as Environmental Science, Chemistry, Chemical Engineering and Material Sciences which is around 68.8%. Thus, it depicts that there is a need for the present study of adsorptive removal of phenolic pollutant. Over a period, several methods are developed to remove phenolic pollutants from wastewater.

Some of the prominent technologies are advanced oxidation process, ion-exchange, solvent extraction, biodegradation, photo-degradation, membrane filtration and adsorption (Awual et al., 2013; Hena et al. 2016; M. Kumar et al. 2018; P. Kumar et al. 2020; Nassar et al. 2014; Zhu et al. 2019). One of the disadvantages of these methods except adsorption is failure to completely remove the phenolic pollutants from effluent (Bentaleb et al. 2017). So, adsorption is considered one of the best and well-established methods for treatment of pollutants. The added advantage of using adsorption technique is implementation, cost-effectiveness and efficiency as compared to other methods. The literature data corroborates that adsorption was performed using clays, sludge (Aksu and Gönen 2006), zeolite (Yousef and El-Eswed 2009), fly ash (Bandura et al. 2015) and alumina (Liang et al. 2015). With evolvement, activated carbon (AC) (Mohammadi et al. 2020), biochar (Jain et al. 2022), nanoparticles/nanocomposites (Zong et al. 2020) and metal organic frameworks (MOFs) (Mohd Azmi et al. 2020) were also considered for the various studies involving pollutant removal.

However, activated carbons derived from biomass/agricultural waste are extensively used due to plenty of source available for synthesis. AC from wood, groundnut shell and coconut shells etc. are few of the commercially available sources (Eletta et al. 2020; Ho and Adnan 2021; A. Kumar et al. 2022; Wahid et al. 2022), though there are several other sources such as shells of almond, walnut and arecanut that could be considered carbon precursors (Lalhmunsiama et al. 2017; Kai Li et al. 2018; Xie et al. 2020). Few of the recent studies made use of cow-dung (Jain et al. 2022), wheat straw (Shi et al. 2022), bamboo sheath (Ezung et al. 2022), sugarcane bagasse (Greish et al. 2021) etc. for remediation of phenolic pollutants. There are a wide range of agricultural wastes that serve as precursor of carbon. However, the properties and yield of the activated carbon are a function of the type of source, impregnation ratio (IR), reagent used, activation temperature and duration. Many of the researches involved in studying effect of these variables are discussed further. To begin with, several investigators have used coconut, blackwattle bark waste (Ho and Adnan 2021; Lütke et al. 2019) with ZnCl_2_ (1:1 to 1:20) as activating agent. Further, bamboo and *Tithonia diversifolia* (Ezung et al. 2022; Supong et al. 2020) treated with KOH (1:2) were also used for phenol adsorption. Furthermore, in certain recent findings, Foxnut shell and oakwood treated with H_3_PO_4_ (1:1.5–1:2) were also used (Dehmani et al. 2022; A. Kumar et al. 2022). Comparable investigations were carried out utilising prickly pear seed cake and *Artocarpus champeden*, employing H_3_PO_4_ as activating agent, resulting in the production of activated carbon with yield ranging from 45 to 60% (Bijang 2022 Dhahri 2022). Further, the working condition especially higher activation temperature greater than 500°C with same activation agent resulted in lesser yield say around 30–35% (Yakout and Sharaf El-Deen 2016). Similar results were also reported for mangrove-based carbon (Zakaria et al. 2021; Jamalluddin, and Abu Bakar 2021). From the available literature, it was evident that considerable research was accessible on usage of orthophosphoric acid as a activating agent (Ren et al. 2011) using biomass waste. So, *Cassia fistula* pods, a locally available biomass was chosen for further studies.

*Cassia fistula* (CF) trees are a habitat of dry and moist deciduous forests that could be used as source of biomass for our studies. They are native of India and Sri Lanka commonly called as Indian Laburnum, Purging fistula, Golden shower and locally called as Kakke mara. In India, the species is widely distributed in Andhra Pradesh, Kerala, Odisha, Tamil Nadu and Arunachal Pradesh and in selected portions of Karnataka as well. The non-edible fruit/pod of CF is dark brown coloured and cylindrical in shape. The pod has a shell covering the sticky brown pulp. These pods are unused and consequently plenty of them are wasted every year. Therefore, this confirms that CF pods can be employed effectively as an affordable, naturally available material for the development of activated carbon. As per our knowledge, no detailed information about developing mesoporous activated carbon using *Cassia fistula* (CF) is available in literature. Further sorption potential of CF-derived activated carbon on removal of organic pollutants has not been published. Herein, a new high surface area adsorbent which was developed using simple steps is reported. In this article, (i) details of synthesis and characterisation of *Cassia fistula*–based activated carbon; (ii) optimisation of operational parameters for phenol and 2,4-DCP; (iii) isotherm-modelling, kinetics and thermodynamics (isosteric heat of adsorption); (iv) mechanism involving adsorption of phenol/2,4-DCP; and (v) desorption/regeneration studies are discussed.

## Materials and methods

### Chemicals required

Analytical grade reagents phenol (C_6_H_5_OH), 2,4-dichlorophenol (C_6_H_4_OCl_2_), sodium bicarbonate (NaHCO_3_), orthophosphoric acid (H_3_PO_4_), sodium hydroxide (NaOH), hydrochloric acid (HCl) and sodium chloride (NaCl) were procured from Merck India Ltd., AR grade.

### Preparation of adsorbate and activated carbon

#### Preparation of synthetic wastewater and activated carbon

Initially, 1 g of phenol was added to 1000 ml of double distilled water to prepare 1000 mg/L phenol solution. The mixture was stirred till solid crystals of phenol are completely dissolved.

A procedure as documented by Zhang et al. (2021) was followed with slight modifications. To prepare activated carbon, initially, the locally available raw biomass, i.e. *Cassia fistula* (CF), was obtained from Manipal Region, Karnataka. The pods were cleansed with distilled water and allowed to dry in the oven for 24 h at 105°C. The shell of the dried *Cassia fistula* pod was separated and pulverised. The powdered sample passing through sieve (< 425 μm) was collected and allowed to dry for another 24 h (Lütke et al. 2019). Then, the fine dried sample was mixed with H_3_PO_4_ (1:1 ratio) and allowed for reaction. The impregnated sample was then placed in furnace at 500 °C for 60 min in an inert atmosphere. Then, activated sample was allowed to cool and further washed with 1–2% NaHCO_3_ solution till the pH raised to 7. Then, the washed sample was oven-dried for 24 h, labelled as CFPAC and stored in an airtight box.

### Characterisation of CFPAC

#### Proximate and ultimate analysis

The proximate analysis was carried out as per IS 1350 (Part-I) (Jadhav and Srivastava 2013) and ultimate analysis (CHNS) was investigated (ELEMENTAR Vario EL III).

#### Surface area, pore volume and XRD

Surface area and pore volume of CFPAC were investigated by Brunauere-Emmette-Teller (BET) technique (Autosorb IQ-XR-XR, Anton Paar, Austria) (Bibaj et al. 2019). The X-ray diffractometer (XRD) study was performed (Rigaku Miniflex 600 5th gen), for 2θ values ranging from 5 to 80°. The data of various peaks pertaining to the sample were obtained, and a graph of 2θ vs intensity was plotted to comprehend crystallographic structure of CFPAC.

#### Scanning electron microscopy-EDS and FTIR

The magnified images of the sample were obtained from scanning electron microscopy (SEM) (VEGA3 TESCAN). These images help in understanding surface morphological characteristics of CFPAC (Osasona et al. 2022). Further, the occurrence of different elements present in CFPAC was obtained from energy-dispersive x-ray spectroscopy (EDS) (Ramutshatsha-Makhwedzha et al. 2022).

Infrared spectroscopy (ATR-FTIR Shimadzu-8400S) of a CFPAC  was carried out to identify the prominent functional groups. The Fourier transform (FTIR) spectroscopy is one of the common techniques used for this purpose. The method allows analysis of molecular vibrations by measurement of absorption of infrared rays, by which it reveals a distinct pattern of spectra (ATR). The spectra is associated with the functional group which is unique for materials (Sanjeev et al. 2023; Vallabha et al. 2023).

#### TGA study

To understand the stability of prepared adsorbent, thermogravimetric analysis (TA 55 discovery, TA instruments Austria) was incorporated for CFPAC mass of 5 mg by heating from 26 to 800 °C. The procedure further required maintaining a constant rate of heating at 10 °C per min with N_2_ supply of 20 mL/min at various temperature (Lütke et al. 2019).

#### Point of zero charge (pH_PZC_)

The pH_PZC_ is the point or pH at which charge on the adsorbent is neutral. In other words, the pH at which net positive functional groups are equal to net negative functional groups is called pH_PZC_. Drift method was followed as mentioned by Gonçalves Júnior et al. (2022). Initially, 500 mL of 0.01N NaCl solution was prepared and 50 mL of the salt solution was transferred to six different conical flasks. The pH of the solution was altered from 2 to 12 using dilute solution of acid and base. 50 mg of adsorbent was taken in each flask and kept for agitation for 24 h. The change in pH was noted and plot of pH_initial_ vs pH_final_ was plotted.

### Optimisation of operational parameters

#### Effect of operational parameters

The operational parameters such as contact time (0–180 min), dosage (0.2–4 g/L), initial concentration (25–600 mg/L), pH (2–12), temperature (20–50 °C) and agitation speed (50–200 rpm) were optimised. The pH of solution (2–12) was varied using standard acid and base solution of HCl and NaOH (0.1–1 N) (Mandal and Das 2019). The experiments were conducted in a 250mL standard conical flask with a stopper; working volume (V) of 50 mL for varied initial concentration (C_o_) as mentioned above. Initially, the study was performed with an adsorbent dosage (m) of 50 mg, working speed of 150 rpm, temperature at 30°C in thermostat till equilibrium was reached. Once equilibrium was attained, the samples were collected in clean, dry vials. Further, samples are centrifuged to reduce interference of particles in the sample and analysed using a UV–Visible Spectrophotometer (UV 1900i, Shimadzu, Japan). The removal efficiency and adsorption capacity of pollutant were calculated using Eq. 1 and Eq. 2. Similar studies were also executed for 2,4-DCP. The optical density of phenol and 2,4-DCP was detected at a wavelength of λ_max_ = 270 nm and 284 nm respectively. All the optimisation experiments were accomplished in triplicates.

1 $${\mathrm{\%Removal}}_{({\text{Phenol}}/\mathrm{2,4}-{\text{DCP}})}=\left(\frac{{C}_{{\text{o}}}-{C}_{{\text{e}}}}{{C}_{{\text{o}}}}\right)*100$$

2 $${q}_{{\text{exp}}}=\left(\frac{{C}_{{\text{o}}}-{C}_{{\text{e}}}}{m}\right)*V$$

where *C*_o_ is the initial concentration of phenol/2,4-DCP (mg/L) and *C*_e_ is the equilibrium concentration (mg/L), *V* is the volume of pollutant (mL), and *m* is the dosage of CFPAC (mg) and *q*_exp_ denotes the experimental adsorption capacity (mg/g).

### Isotherm, kinetics and modelling

#### Isotherm studies

The isotherm and kinetic studies for phenol and 2,4-DCP were performed at optimised conditions. In isotherm studies, phenol solution of varied initial concentrations ranging between 25 and 600 mg/L under the optimum conditions was performed. The experimental data was fitted to isotherm models i.e. Langmuir isotherm, Freundlich isotherm, Temkin model and Redlich-Peterson-model. The details of isotherm models are listed in the supplementary file Table S2.

#### Kinetic studies

In kinetic studies, samples were collected initially at short interval of time and later the interval was increased till equilibrium is attained. Further, the collected samples were centrifuged, and absorbance of each sample was measured in UV–Visible spectrophotometer (UV-1900i, Shimadzu). The obtained absorbance was further analysed andconcentration with respect to time was calculated. The data obtained from kinetic studies were modelled using linear and non-linear forms of equations (Abdulrahman et al. 2023; Kunwar et al. 2023). To compute the activation energy (*E*_a_, kJ/mol) of the adsorption process, kinetic studies at varying temperatures (20–50°C) was also performed. Arrhenius equation was utilised to assess the *E*_a_ of adsorption, which corresponds to the minimal energy essential for reaction to progress. The relationship is mathematically represented as below.

3 $${{\text{lnk}}}_{2}={\text{lnA}}-\frac{{{\text{E}}}_{{\text{a}}}}{{\text{RT}}}$$

*k*_2_ (g/mg min) corresponds to the rate constant (second order), *E*_a_ (kJ/mol) denotes the Arrhenius activation energy of adsorption and *A* stands for the Arrhenius factor. When ln (*k*_2_) vs 1/*T* is plotted, a linear relationship with slope of − *E*_a_/*R* is obtained. The value of *E*_a_ was further calculated using the linear form by substituting the value of *R*.

Further, to decide the rate limiting step of adsorption process, Weber and Morris/intraparticle diffusion model was also used. The specifics of the kinetic models employed in the study are given in supplementary file Table S3.

### Thermodynamic investigation

#### Thermodynamic parameters and isosteric heat of adsorption

The change in important thermodynamic parameters namely standard enthalpy (Δ*H*°), standard entropy (Δ*S*°) and standard free energy (Δ*G*°) due to transfer of unit mole of solute from solution onto the solid–liquid interface is considered vital in thermodynamics of adsorption process. Additionally, *E*_a_ is a key factor in this context. The detail regarding *E*_a_ is discussed in the preceding subsection. For this purpose, the adsorption of pollutant on CFPAC was carried out at different temperatures ranging from 20 to 50°C. The adsorption of each initial concentration, say from 25 to 600 mg/L onto CFPAC, was studied in batch mode. The value of Δ*H*° and Δ*S*° can be computed using the following equations as given below:

4 $${{\text{ln}}K}_{{\text{L}}}=\frac{\Delta {S}^{{\text{o}}}}{R}-\frac{\Delta H^{o}}{{\text{RT}}}$$

To estimate Δ*H*° and Δ*S*°, one needs to determine the slope and intercept of the van’t Hoff plot, where ln *K*_L_ is plotted against 1/*T*. Δ*G*° can then be calculated using the relation below:

5 $${\Delta G}^{{\text{o}}}=\mathrm{-RT*lnK_{L}}$$

Further, for each concentration, the graph of ln (*K*_T_) vs 1/*T* was plotted. The equilibrium constant is given by,

6 $${K}_{T}=\frac{{C}_{{\text{s}}}}{{C}_{{\text{e}}}}$$

where *C*_s_ is defined as the adsorbate present in solid phase and can be calculated as difference of initial concentration (*C*_o_) and equilibrium concentration (*C*_e_), and *T* in absolute temperature in Kelvin.

Further, isosteric heat of adsorption was also investigated. The procedure involves calculating the said parameter (Δ*H*_x_) at constant adsorbed pollutant (say *q*_e_ = 10–110 mg/g for phenol and *q*_e_ = 40–390 mg/g for 2,4-DCP) using Clausius–Clapeyron equation as given below.

7 $${{\text{ln}}(C}_{{\text{e}}})=-\frac{{\Delta H}_{{\text{x}}}}{{\text{RT}}}+Constant$$

The equilibrium concentration (*C*_e_) values for the constant adsorbed quantity (phenol and 2,4-DCP) were deduced from equilibrium studies data conducted from lower to higher operational temperatures ranging from 20° to 50 °C. The importance of conducting this investigating lies in its potential to elucidate, if the process of any adsorption is dominated by physical/chemical type (Singh, Kumar, and Kumar 2016). Moreover, this study can be regarded as validation of the thermodynamic investigation.

#### Spiked studies on water samples (phenol and 2,4-DCP)

The efficacy of the CFPAC was also tested by spiking real-time water samples with phenolic pollutants to understand the practical applicability of the adsorbent (Mohammadi et al. 2020). Grab samples were collected in a clean container from nearby waterbodies such as sea water, well water and tap water. The adsorption study was performed under optimised conditions.

#### Cost analysis for production of CFPAC

The cost analysis for the preparation of activated carbon was quantified at lab scale. The analysis offers a comprehensive understanding of economic implications related to production phase. By analysing the resource used, breakdown of cost would be possible to save expenditures. The study will not only benefit in terms of cost but also with sustainability. The details include cost of chemicals, electricity, deionised water and other utilities (Vukelic et al. 2018).

#### Desorption and regeneration study of CFPAC

Initially, the CFPAC was loaded with phenol and the experiment was run till equilibrium (3 h). Later, aqueous solution of the phenol was separated. The adsorbent was allowed to dry and further the desorption batch study was performed in eluent, in alkaline (0.1N NaOH) condition (Sathya Priya and Sureshkumar 2020). Further, adsorbent was separated from eluent, washed and allowed to dry. The dried adsorbent was used for next adsorption cycle. The %desorption was calculated using the equation below where C_d_ is the concentration of desorbed pollutant,

8 $$\mathrm{\%Desorption}=(\frac{{C}_{{\text{d}}}}{{C}_{{\text{o}}}-{C}_{{\text{e}}}})*100$$

## Results and discussion

### Characterisation of CFPAC

#### Surface property, proximate and ultimate analysis

Fig. S1 illustrates N_2_ adsorption/desorption isotherm for CFPAC. The obtained profile corresponds to type IV isotherm accompanied by hysteresis indicating mesoporous material as per IUPAC (MacHado et al. 2020). The surface area and pore volume of CFPAC was approximately 1146 m^2^/g and 0.8828 m^3^/g. The obtained parameters substantiate higher surface area and volume which are comparable to recent investigations (Abdulrahman et al. 2023; Allahkarami et al. 2023).

Further, proximate analysis of CFPAC resulted in high fixed carbon (55.125%) and lower ash content (3.385%) (Boontham et al. 2020) and ultimate analysis of CFPAC revealed presence of C (49%), H (7.41%), N (0.86%) and S (0.07%). These surface properties would be more promising for adsorption studies. Additionally, the data of proximate and ultimate analysis gives a positive indication on the process followed for the development of CFPAC (details enlisted in Table S4 of supplementary data).

#### Analysis of functional groups

**Fig. 1:**
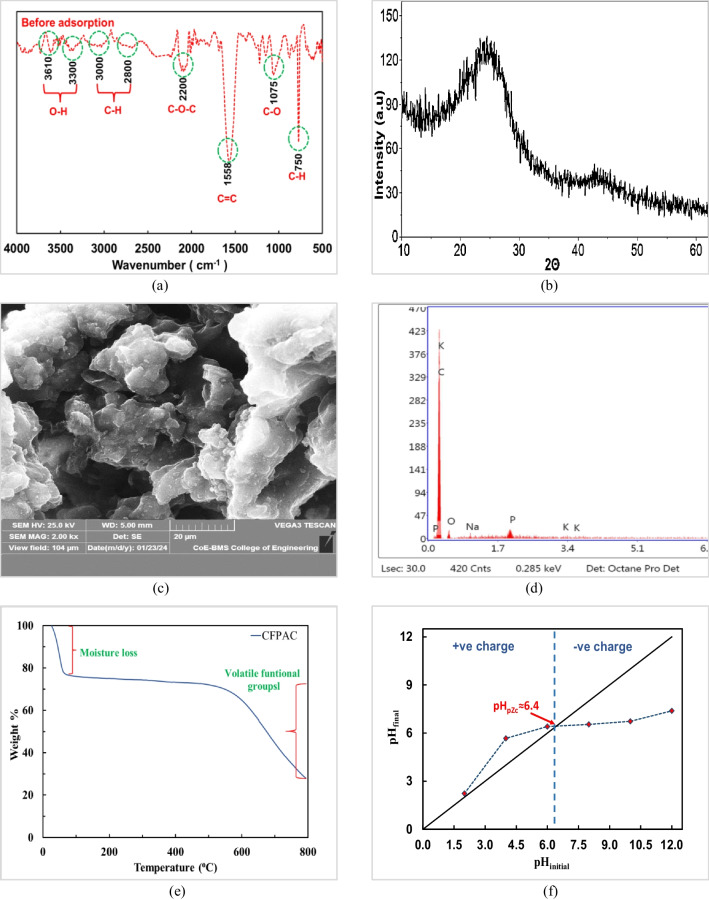
Characterisation of CFPAC: **a** FTIR, **b** XRD, **c** SEM at 2 K magnification, **d** EDX, **e** TGA and **f** pH_PZC_ analysis

The FTIR spectra of CFPAC are shown in Fig. 1a. The spectra illustrates that there are multiple funtional elements are detected. This was mainly due to thermal and chemical activation. Two peaks in the region 3250–3650 cm^−1^ at 3300 and 3610 cm^−1^ are due to O–H stretching of adsorbed water, alcohols and carboxyls. Further, the peaks between 2700 and 3100 cm^−1^ are due to stretching of C–H bonds of alkanes and alkyls groups (Vohra et al. 2023). The peak at 2200 cm^−1^ depicts C–O–C streching of esters (Nwabanne et al. 2022) and the peak present at 1558 cm^−1^ is assigned to stretching of C = C aromatic rings. Further, the peak between 1000 and 1200 cm^−1^ represents stretching of acids, ethers and esters while peak at 750 cm^−1^ represents stretching of aromatic C–H bond. These funtional groups can play a significant role in removal of phenolic pollutants.

#### XRD and SEM–EDS analysis

The broad peak between 13° and 30° at 2θ ≈ 25° (002) and inferior peak between 40° and 48° at 2θ ≈ 45° (100) plane are observed. These peaks correspond to carbon and the wide range of diffraction peaks depict that the activated carbon was amorphous as given in Fig. 1b. Moreover, it is also associated with the presence of “carbon graphite” (Franco et al. 2021). The obtained peaks were alongside the standard XRD plots as reported in previous studies (Ezung et al. 2022; Shi et al. 2022; da Silva et al. 2022). In general, it is viable to state that adsorbent with disordered/disorganised aromatic structural arrangements thus indicate amorphous material. For the further investigations, amorphous materials may be promising for the adsorption phenomena given that there would be more pores and space for adsorption (Gonçalves Júnior et al. 2022). Analogous findings were reported for pomegranate husk–derived biochar (Taheri et al. 2022).

The structural characteristics of CFPAC were investigated by SEM at 2 K magnification as shown in Fig. 1c. From the magnified picture, it can be observed that, asymmetric, oval shape of pores on CFPAC are developed of the magnitude 20 µm. The images also depicted an ample number of cracks which was due to calcination at 500°C (Tao et al. 2019). Thus, with increased pore entries, there could be increased functional groups. These openings would be advantageous for enhancing adsorption capacity of phenol and 2,4-DCP (Osasona et al. 2022). Comparable results were also reported in the research carried out by Abdulrahman (Abdulrahman et al. 2023). Figure 1d also demonstrates EDS spectra of CFPAC representing distribution of various elements. The elements such as C (90.65%), O (7.735%), Na (0.28), Si (0.08%) and P (1.19%) are present (details provide in supplementary data Table S5). The %C was relatively very high as compared to findings reported by Tao (Tao et al. 2019). Further, the absence/least presence of other alkali/alkaline earth metals portrays that the activation and washing steps were successful. The presence of a small fraction of phosphorus depict usage of orthophosphoric during the preparation of CFPAC (Samanth et al. 2023). Further, Fig. S10(a) and (b) illustrates magnified images post-adsorption of phenol molecules (0.7 k) and 2,4-DCP (1 k) onto activated carbon. From both the images, it can be observed that the size of pores was diminished to approximately < 20 µm. In addition, a change in intensity of carbon (C)  Fig. S10(a) and (b) depicts utilisation of carbon for adsorption of phenol. Furthermore, from the elemental composition as listed in Table S5, it was found that the mass fractions of chlorine (Cl) surged from 0 to 3.045%. This suggests and confirms successful adsorption of 2,4-DCP onto the surface of CFPAC. Thus, these findings are indicative of effective adsorption of both pollutants.

#### Thermogravimetric analysis

Thermogravimetric profile of CFPAC is shown in Fig. 1e. The initial weight loss (approximately 22%) till 100°C was mainly attributed to dissipation of moisture content. Furthermore, the losses (approximately 44%) between 550° and 800°C could be attributed to loss of volatile components present in CFPAC.

#### Point of zero charge (pH_PZC_)

The point of zero charge was performed by following method as described by the authors Kuśmierek et al. (2016) and Zhao et al. (2023). From Fig. 1f, the obtained pH_PZC_ value of 6.4 implies that, adsorbent bears net positive charge when pH_PZC_ > pH_solution_ and negative charge if pH_PZC_ < pH_solution_. Comparable results were also documented for the removal of phenol using Bamboo and Brazil nut (Pei et al. 2023a; da Silva et al. 2023). In other words, it could also be said that, for the present study, acidic pH would favour adsorption of adsorbate (Dehmani et al. 2022).

### Optimisation of operation parameters for phenol and 2,4-DCP adsorption

#### Effect of contact time

Initially, both the pollutants were tested to arrive at the equilibrium time required for the conduction of batch experiments. For this purpose, 25 ppm of phenol and 50 ppm of 2,4-DCP which were considered certain dosage of prepared adsorbent were tested till saturation time was reached. From Fig. 1a, it could be concluded that the equilibrium time for phenol and 2.4-DCP was 180 min and 120 min, respectively.

#### Effect of dosage and initial concentration

**Fig. 2:**
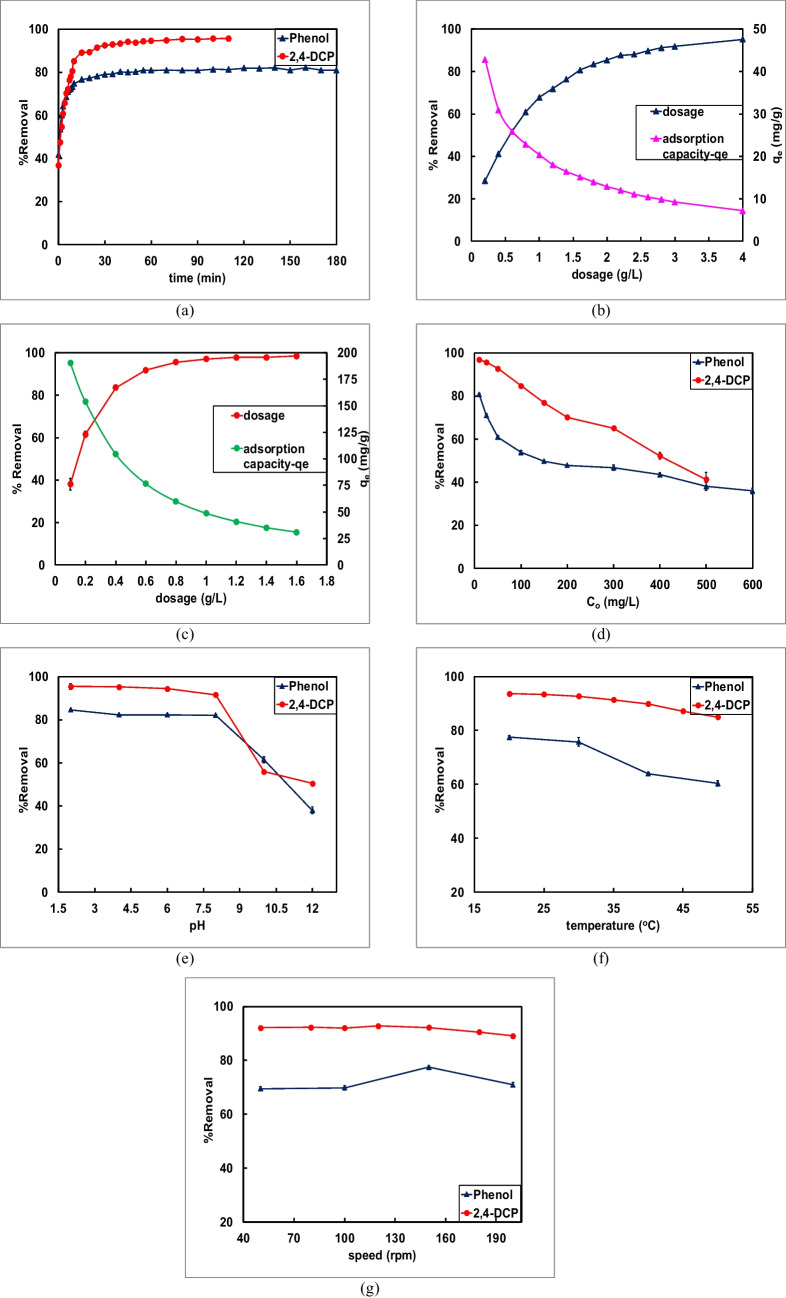
Effect of operational parameters on removal of phenol[P] and 2,4-DCP: **a** time; **b**, **c** dosage; **d** concentration; **e** pH; **f** temperature; and **g** agitation speed. (Experimental conditions (**a)** *t*, 0–180 min; pH, 7; CFPAC dose, 1.6/0.6 g/L; P/2,4-DCP conc., 30/50 mg/L; T, 25°C; speed, 150 rpm; (**b)** CFPAC dose (0.2–4 g/L), t, 0–180 min; pH, 7; P conc., 30 mg/L; T, 25°C; speed, 150 rpm; (**c)** CFPAC dose (0.2–1.6 g/L); t, 0–120 min; pH, 7; 2,4-DCP conc., 50 mg/L; T, 25°C; speed, 150 rpm; (**d**) P/2,4-DCP conc., 25–600 mg/L; CFPAC dose 1.6/0.6 g/L; t, 180/120 min; pH, 7; T, 25°C; (**e**) pH, 2–12; P/2,4-DCP conc., 25/50 mg/L; CFPAC dose 1.6/0.6 g/L; *t*, 180/120 min; *T*, 25°C; (**f**) *T*, 20–50°C; P/2,4-DCP conc., 25/50 mg/L; CFPAC dose 1.6/0.6 g/L; *t*, 180/120 min; pH, 2; (**g**) speed, 50–200 rpm; P/2,4-DCP conc., 25/50 mg/L; CFPAC dose 1.6/0.6 g/L; *t*, 180/120 min; T, 20°C; pH, 2)

Figure 2b and c display impact of varying dosage on removal of phenol and 2,4-DCP. Batch tests were accomplished to investigate impact of adsorbent dosage (g/L) in the adsorption process. In case of phenol, the removal percentage increased from 27.98% to roughly 95.21%; meanwhile, the adsorption capacity dropped to 7.14 mg/g from 41.98 mg/g as shown in Fig. 2b. Similarly, with reference to 2,4-DCP, ascend of percentage removal from 38.09 to 98.48% for the dosage change from 0.1 to 1.6 g/L was observed, despite that adsorption capacity declined to 30.7 mg/g from 190.4 mg/g as illustrated in Fig. 2c. The rise in removal was due to availability of excess active adsorption sites due to increase in dosage that was added. However, the dip in adsorption capacity was due to the inverse relation of adsorption capacity and dosage (Eq. 2). This was also because, with rise in dosage, several sites responsible for adsorption remain unoccupied which resulted in surplus dosage and hence drop in adsorption capacity. In the context of phenol adsorption, the point of intersection of percentage removal and adsorption ability was supposed to be finalised as optimum dosage (0.8 g/L) (Vinayagam et al. 2023). However, the removal was only 60%. Therefore, a higher dosage of 1.6 g/L was chosen as optimum dosage. The dosage not only provided higher removal of 80.2% but yielded a good adsorption capacity. Likewise, 0.6 g/L of CFPAC was finalised as optimum dosage for treatment of 2,4-DCP at which a higher removal percentage (91.86%) and adsorption capacity (76.55 mg/g) was possible.

Figure 2d displays the effect of concentration of phenol and 2,4-DCP. Batch studies were conducted using CFPAC for various initial phenol (25–600 mg/L) and 2,4-DCP (25–500 mg/L) amounts till 3 h by adjusting operational parameters (speed, temperature) unaltered (Yan et al. 2019). In both the cases (Fig. 2d), there was decline in percentage removal with increase in adsorbate concentration. The fall was due to inadequacy of sites on CFPAC for elevated phenol and 2,4-DCP concentration (Mandal and Das 2019). For further studies, 25 mg/L of phenol and 50 mg/L of 2,4-DCP were chosen owing to higher percentage removal.

#### Effect of pH and temperature

The influence of pH_solution_ on adsorption was performed and standard acid (HCl) and base (NaOH) solution were used to alter the pH. From Fig. 2e, the highest removal efficiency was reflected at pH 2. Further, the change in percentage removal from pH 2–8 was trivial. The obtained pH_PZC_ was approximately 6.4 as shown in Fig. 2e. So, when pH_solution_ < pH_PZC_, the phenol molecule is attracted towards positively charged CFPAC and thus adsorption favours due to electrostatic attraction. Unlike at pH_solution_ > pH_PZC_, small molar fraction of phenol starts transforming into phenolate, and adsorbent develops a negative charge. Subsequently, due to the electrostatic repulsion between adsorbate and adsorbent molecule, a sharp descent in removal of phenol can be observed after pH 8 (da Silva et al. 2023). Further, phenol acts as weak acid with pK_a_ value 9.89 and tends to dissociate when pH_solution_ > pK_a_. Due to this reason, the adsorption quantity leads to subside after pH 8 due to ionisation of aqueous phenol. The results were similar to the one reported by Kumar for the adsorption of phenol (A. Kumar et al. 2022). So, in further studies, pH 2 was chosen as optimum pH.

Further, the impact of pH_solution_ on adsorption of 2,4-DCP was also investigated. There was minor descent in removal of 2,4-DCP when pH was altered from 2 to 8, the highest being at pH 2. However, from pH 10, a slightly more noticeable drop in the removal was examined as observed from Fig. 2e. This behaviour of 2,4-DCP at altered pH indicates that there could be certain interaction between pollutant and CFPAC surface. Similarly, a trend was also observed in the research carried out by Shaarani and Taheri (Shaarani and Hameed 2010; Taheri et al. 2022). The pH_PZC_ of CFPAC was about 6.4 and pK_a_ of 2,4-DCP nearly 7.48 as obtained from literature. The net charge on CFPAC is positive when pH_PZC_ > pH_solution_ and negative charge if pH_PZC_ < pH_solution_ (Gonçalves Júnior et al. 2022). The sudden descend in efficiency at pH > 8 could be described by electrostatic repulsion of same charge (negative) of adsorbent and pollutant. In addition, at pH_solution_ > pK_a_, the 2,4-DCP molecule tends to exist in the anionic state and the dissociation keeps rising with higher pH. In acidic pH, a higher number of protons are available, thus favouring electrostatic type of attraction between molecular form of 2,4-DCP and positively charged CFPAC and hence increasing efficiency of adsorbent. However, in alkaline condition, the pollutant molecule started dissociating into negatively charged chlorophenolate ions making it unfavourable for adsorption as the CFPAC surface was either negative or neutral (Kuśmierek et al. 2016; Song et al. 2023). Therefore, for further studies, pH 2 was considered optimum for further studies.

Figure 2f illustrates influence of temperature ranging between 20 and 50°C on phenol and 2,4-DCP removal. In the case of phenol, the result signifies that percentage removal was highest at 20°C and decreased with rise in temperature till 50°C. This phenomenon might have arisen due to change in chemical interactions between the surface functionality of CFPAC and the phenol molecule. The result also points out depicting exothermic adsorption process (Mandal and Das 2019). Figure 2f corroborates that temperature also has significant influence on adsorption of 2,4-DCP on CFPAC. With rise in temperature, the molecules attain additional kinetic energy to overcome adsorption forces and thus escape or do not bind to CFPAC surface. It also indicates exothermic reaction occurring during the process of adsorption of 2,4-DCP (Akhtar et al. 2006). Analogous trend has been reported by Kumar and Medellin in their research work (A. Kumar et al. 2022; Medellín-Castillo et al. 2021). Thus, 20°C was finalised as optimum temperature for further studies.

#### Effect of agitation speed

Agitation speed in adsorption is also considered one of significant operating parameters. The impact of different shaking speeds on adsorption of phenol on CFPAC was performed. The details are provided in Fig. 2g. It can be noted that there was minor change of 0.3% removal between 50 and 100 rpm agitation speed. However, a significant surge was observed at 150 rpm. This was since the agitation speed had a positive convective mass transfer effect by shrinking the boundary layer closest to adsorbent. The findings from the plot also depict that phenol molecules were effectively transferred to the surface/pores of CFPAC due to decline in diffusion resistance at 150 rpm rotational speed. However, at 200 rpm, there was a fall in removal percentage. This was because pollutant molecules tend to escape/desorb from pores or there could be chances of abrasion of CFPAC at higher speed. Thus, 150 rpm was considered the ideal speed for future experimentations (A. Kumar et al. 2022).

Similarly, impact of operational speeds ranging between 50 and 200 rpm was also investigated on adsorption of 2,4-DCP. The details can be observed in Fig. 2f. There was trivial uptrend of 0.69% removal till 120 rpm. However, a significant drop of 3.67% after 120 rpm was observed facilitating diffusion of pollutant molecules into pores of CFPAC. It can be inferred that the effective transfer of 2,4-DCP ions to the surface of CFPAC occurred as a result of a decrease in diffusion resistance at a higher rotational speed (120 rpm). Similar agitation speed was also used in for adsorption studies by Song (Song et al. 2023). Hence, 120 rpm was chosen as the optimal speed for further investigations.

### Isotherm studies and modelling

Once all the parameters are optimised, two- and three-parameter isotherm model as expressed in the Table S2 are fitted to experimental data. The optimised condition chosen to perform further isotherm studies are listed in Table S6 of supplementary file.

Herein, experimental data obtained at pH 2 and pH 5 were modelled and fitted to isotherm models to comprehend mechanism of adsorption and to understand the variation in the adsorption capacities (Fig. S2). The isotherm studies for phenol adsorption on CFPAC demonstrated better fit in most of the cases as observed from *R*^2^ values (Table 1). However, the order of fit was Redlich-Peterson > Freundlich > Langmuir > Temkin for the isotherm studies performed at pH 2 (Cheng et al. 2016; Ta et al. 2021) with trivial changes at pH 5. At both pH, Redlich-Peterson isotherm suitably justified the adsorption process. Moreover, the monolayer adsorption ability for phenol at pH 2 was 183.79 mg/g and at pH 5 was 149.76 mg/g. The data from Table 1 corroborates that three-parameter model was better as compared to two-parameter isotherm model (Ta et al. 2021). As we have considered higher adsorbate concentration and β approaches zero at pH 2, it can be inferred that the adsorption process was more accurately described by Freundlich model (Cheng et al. 2016) with *R*^2^ = 0.9964. Conversely, at pH 5, β tends to one, thus establishing Langmuir isotherm with *R*^2^ = 0.9859, better describing the process.

**Table 1:** Equilibrium parameters for adsorption of phenol and 2,4-DCP on CFPAC

Pollutants	Model	Constants	Units	pH 2	*R*^2^	SSE	*χ*^2^	Equation	pH 5	*R*^2^	SSE	*χ*^2^	Equation
Phenol	Langmuir	*q*_*m*_	mg/g	183.79	0.9876	188.34	0.2992	${q}_{e}=\frac{1.1597{C}_{e}}{1+0.0063{C}_{e}}$	149.76	0.9859	123.78	0.2691	${q}_{e}=\frac{1.028{C}_{e}}{1+0.00687{C}_{e}}$
		*K*_*L*_	L/mg	0.0063					0.0068				
	Freundlich	*n*	constant	1.917	0.9964	55.26	0.0856	${q}_{e}=6.11*{C}_{e}^{0.5216}$	2.124	0.9661	167.06	0.3553	${q}_{e}=6.787*{C}_{e}^{0.471}$
		*K*_*f*_	mg^1−n^L^n^/g	6.110					6.787				
	RP-model	*K*_*RP*_	L/g	21.38	0.9969	46.49	0.0784	${q}_{e}=\frac{21.38{C}_{e}}{1+1.758{C}_{e}^{0.495}}$	2.043	0.9982	103.3	0.2111	${q}_{e}=\frac{2.043{C}_{e}}{1+0.782{C}_{e}^{0.733}}$
		*µ*	L/g	3.132					0.782				
		*β*	constant	0.495					0.733				
	Temkin model	*b*_*T*_	J/mol	98.54	0.8960	1575.85	2.3752	${q}_{e}=24.72{\text{ln}}(0.27{C}_{e})$	112.78	0.9952	711.31	1.5269	${q}_{e}=21.6{\text{ln}}(0.22{C}_{e}$)
		*A*_*T*_	L/g	0.27					0.22				
2,4 DCP	Langmuir	*q*_*m*_	mg/g	374.4	0.9705	2918.9	1.734	${q}_{e}=\frac{1.1597{C}_{e}}{1+0.0063{C}_{e}}$	344.55	0.9817	1544.38	0.983	${q}_{e}=\frac{1.028{C}_{e}}{1+0.00687{C}_{e}}$
		*K*_*L*_	L/mg	0.045					0.039				
	Freundlich	*n*	constant	3.28	0.9312	6803.7	3.869	${q}_{e}=6.11*{C}_{e}^{0.5216}$	3.08	0.9740	2199.46	1.365	${q}_{e}=5.66*{C}_{e}^{0.5102}$
		*K*_*f*_	mg^1−n^L^n^/g	68.01					55.12				
	RP-model	*K*_*RP*_	L/g	31.28	0.9748	2494.6	1.456	${q}_{e}=\frac{21.38{C}_{e}}{1+3.132{C}_{e}^{0.86}}$	26.032	0.9986	120.97	0.076	${q}_{e}=\frac{0.896{C}_{e}}{1+0.0029{C}_{e}^{0.833}}$
		*µ*	L/g	0.184					0.194				
		*β*	constant	0.86					0.833				
	Temkin model	*b*_*T*_	J/mol	39.80	0.9681	3153.9	1.829	${q}_{e}=61.2{\text{ln}}(0.27{C}_{e})$	40.45	0.9936	537.87	0.336	${q}_{e}=60.21{\text{ln}}(0.22{C}_{e}$)
		*A*_*T*_	L/g	1.168					0.761				

In the case of 2,4-DCP (Table 1), based on *R*^2^ value, the order of fit at pH 2 was Redlich-Peterson > Langmuir > Temkin > Freundlich and at pH 5 Redlich-Peterson > Temkin > Langmuir > Freundlich (Fathy et al. 2022). Further, the monolayer adsorption capacity of 2,4-DCP at pH 2 was 374.4 mg/g and at pH 5 was 344.55 mg/g (Table 1). The disparity in adsorption capacities in both the cases was due to non-availability/less of phenolic compounds for adsorption at pH 5. The detailed explanation of isotherm parameters is explained in the following paragraph. A dimensionless constant separation factor (*R*_L_) can be calculated using Langmuir equilibrium constant (*K*_L_) as below,

9 $${R}_{{\text{L}}}=\frac{1}{1+{K}_{{\text{L}}}{C}_{{\text{o}}}}$$

The separation factor in phenol adsorption decreased from 0.86 to 0.21 (Fig. S3). Likewise, the dip in separation factor for the 2,4-DCP adsorption was observed to be from 0.46 to 0.04 thus depicting favourable and spontaneous adsorption of both the pollutants. Further, a smaller value of *R*_L_ indicates stronger adsorption of pollutant (Nazal et al. 2021).

The obtained values of (1/*n*) were between 0.304 and 0.522 and within the range zero to one describing surface heterogeneity. It can be termed as normal Langmuir, if *n* > 1 and for a value *n* < 1 indicates cooperative adsorption. From RP isotherm, β was found to vary from 0.495 to 0.862 within the specified range of 0 to 1. However, the RP isotherm changes to Langmuir when β approaches unity and transforms to Henry’s law equation when β tends to zero. Further, from Temkin isotherm the value of *A*_T_ varied from 0.22 to 1.168 L/g. A higher value of *A*_T_ depicts that 2,4-DCP adsorption was considerably higher as compared to phenol at same conditions.

**Fig. 3:**
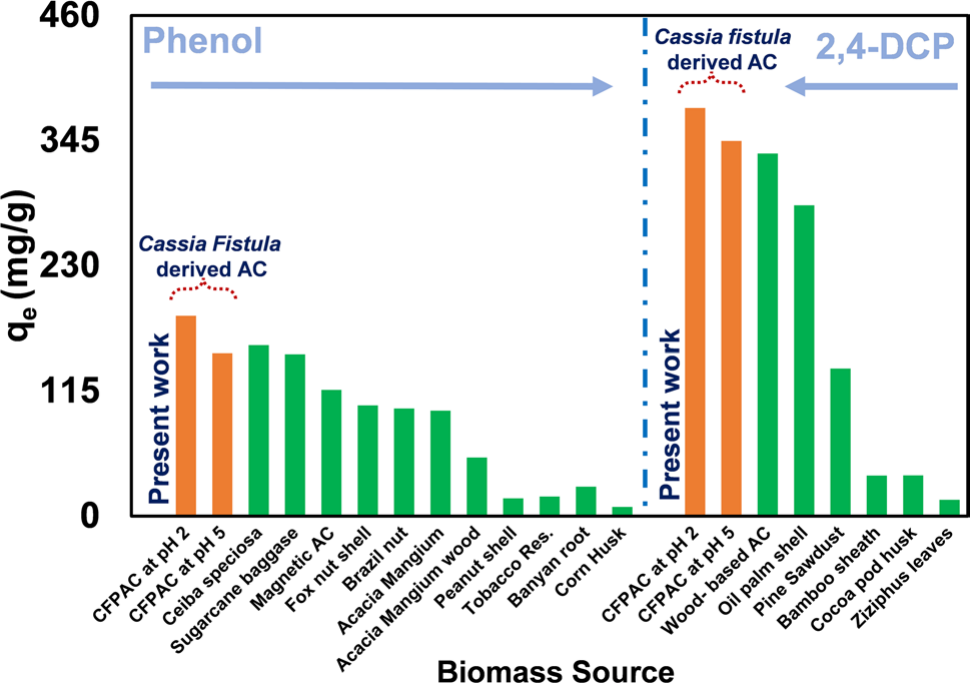
Comparison of adsorption capacity of phenolic pollutant on biomass derived adsorbent

A comparison of monolayer adsorption ability of both phenolic compounds is pictorially represented in Fig. 3 and compared with previous studies (Alam et al. 2023; El-Bery et al. 2022; Ezung et al. 2022; da Silva et al. 2023; Song et al. 2023). It can be depicted that diverse biomass resources are utilised for remediation of phenolic pollutant. Out of all the sources, *Cassia fistula*–derived activated carbon (CFPAC) has given relatively higher adsorption capacity (Fig. 3).

Fig. S4 shows pictorial illustration of Langmuir model for phenol and 2,4-DCP at different temperatures. It can be observed that there is substantial change in the adsorption with increase in operational temperature from 10° to 40°C. A noticeable fall of adsorption capacity by 20% and 43% was observed for phenol and 2,4-DCP respectively when operated at elevated temperature.

### Kinetic study

Adsorption kinetics was carried out, primarily to understand speed of transfer of pollutant molecule and further explore the adsorption mechanism of the phenolic contaminants. Two kinetic models, specifically pseudo-first-order and pseudo-second-order, are investigated. In addition, intraparticle diffusion (IPD) model was also examined.

#### Kinetic model for phenol and 2,4-DCP

The experiments were performed for concentration ranging from 25 to 150 mg/L. The kinetic parameters for phenol and 2,4-DCP adsorption are detailed in Table 2. In both the cases, the *R*^2^_pseudo-second-order_ > *R*^2^_pseudo-first-order_ (Zhao et al. 2023), thus indicating that chemisorption favours the adsorption process of both the pollutants (Kerui Li et al. 2019). Additionally, from the observation, it was found that experimental adsorption capacity was found to be approximately equivalent to adsorption capability obtained from kinetic models illustrating the goodness of the fit. Further, it is noteworthy to mention that 70% of the optimum phenol concentration was instantaneously adsorbed in less than 10 min; however, it reached equilibrium in 180 min. The details can be observed in the non-linear plot shown in Fig. S5(b) and (d). In the case of 2,4-DCP, 90% removal was witnessed in less than 15 min and the system reached equilibrium in 120 min. The findings were less as compared to the previous investigations (Alam et al. 2023; Kilic et al. 2011; da Silva et al. 2023). The details of the linear kinetic plots are provided in the supplementary data Fig. S6.

**Table 2:** Kinetic parameters of phenol and 2,4-DCP adsorption on CFPAC

Pollutant	*C*_o_ (mg/L)	*q*_exp_ (mg/g)	Pseudo-first-order				Pseudo-second-order					IPD model								
			*K*_1_ (min^−1^)	*q*_mod_ (mg/g)	*R*^2^	SSE	*K*_2_ (g/mg min)	*q*_mod_ (mg/g)	*h* (mg/g h)	*R*^2^	SSE	*K*_p1_ (mg/g min^1/2^)	*K*_p2_ (mg/g min^1/2^)	*K*_p3_ (mg/g min^1/2^)	*C*_1_ (mg/g)	*C*_2_ (mg/g)	*C*_3_ (mg/g)	(*R*_1_)^2^	(*R*_2_)^2^	(*R*_3_)^2^
Phenol	25	12.650	0.492	12.31	0.855	10.86	0.0738	12.756	12.008	0.994	0.39	5.768	0.737	0.063	0.000	8.792	12.00	0.995	0.829	0.722
	50	22.385	0.538	21.59	0.823	37.66	0.0453	22.359	22.646	0.989	1.93	10.417	1.294	0.088	0.000	15.505	21.31	0.993	0.868	0.799
	100	38.261	0.594	36.93	0.814	99.57	0.0305	38.156	44.404	0.988	5.48	18.327	2.048	0.144	0.000	27.565	36.44	0.999	0.846	0.731
	150	69.511	1.281	67.63	0.467	192.41	0.0399	69.109	190.56	0.959	18.47	39.992	2.048	0.144	0.000	58.815	67.69	0.974	0.846	0.731
2,4-DCP	25	39.486	0.291	38.26	0.945	75.13	0.0125	40.362	20.36	0.994	6.53	14.531	6.763	0.346	0.000	12.440	36.06	0.999	0.946	0.724
	50	79.725	0.2663	76.80	0.935	402.35	0.0056	81.242	39.69	0.992	42.36	28.828	13.317	0.992	0.000	23.785	70.07	0.998	0.949	0.787
	100	154.004	0.273	144.95	0.913	1887.7	0.003	153.427	70.613	0.989	201.37	56.332	23.018	2.527	0.000	50.640	127.70	0.999	0.951	0.875
	150	210.739	0.266	200.66	0.900	4238.5	0.0021	212.383	94.723	0.981	649.86	79.154	31.844	3.507	0.000	68.558	176.76	0.995	0.960	0.842

The kinetic study at varied temperature can aid in estimating approximate activation energy required for the process. In general, physical adsorption is typically associated with low activation energies (within the range of 5–40 kJ/mol), whereas high activation energies (within the range of 40–800 kJ/mol) are indicative of chemisorption. The details of the plot of kinetics at varying operational temperature are specified in supplementary data Fig. S7. In this investigation, the activation energy (*E*_a_) values for phenol and 2,4-DCP adsorption were 35.62 and 8.5 kJ/mol, respectively. These outcomes suggest that the rate-limiting step in the whole adsorption process could be of a physical nature. Physical adsorption typically occurs at lower temperatures compared to the temperatures associated with chemical adsorption. Additionally, physical adsorption is generally a faster process compared to chemical adsorption, and akin to many reactions, it often involves lower activation energy as investigated in the present case (Baek et al. 2010; Tran et al. 2017).

#### IPD model studies for adsorption of phenol and 2,4-DCP

The IPD model established stages that can reflect the actual adsorption process of phenol and 2,4-DCP. The model is based on Weber and Morris theory. The study was investigated by plotting of *q*_*t*_ vs *t*^1/2^_._ The graph plotted in Fig. S8 depicts that the adsorption process can be fitted into three different segments with straight lines. The first is the sharp region which occurs due to bulk/external diffusion. The second region indicates diffusion from surface to pore and there also exists a third region. The third region depicts the final stage where diffusion of pollutant molecules retards. This is due to less availability of adsorbate molecules at equilibrium.

Referring to Fig. S8(a) and (b), it can be observed that the first stage (external diffusion) was accomplished in approximately 4 min and 2 min in phenol and 2,4-DCP, respectively. The second stage (internal diffusion) was effective for 21 min and 13 min. Further, the third stage (equilibrium stage) was observed for 155 min and 105 min after the second stage. Furthermore, the constant values *C*_2_ and *C*_3_ of stages 2 and 3 substantiate some disparity in mass transfer rates, thus indicating IPD was not the sole rate-limiting step. The details of the rate and other parameters are listed in Table 2. It can be noted that the *K*_p1_ for phenol and 2,4-DCP varied from 5.76 to 39.99 mg/g min^1/2^ and 14.53 to 79.15 mg/g min^1/2^, indicating upsurge in driving force with increasing initial concentration (Kerui Li et al. 2019). Further, in the data for 25 mg/L *K*_p1_ > *K*_p2_ > *K*_p3_ corroborates decrease in rate of adsorption with time and less availability of adsorbate concentration.

### Thermodynamic studies

**Fig. 4:**
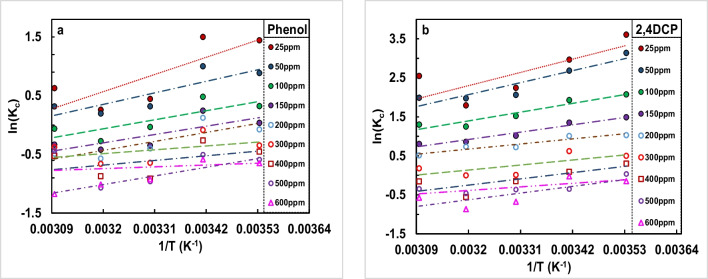
Thermodynamic studies of adsorption of **a** phenol and **b** 2,4-DCP on CFPAC

Herein, the influence of varying temperature (283–323 K) on adsorption of phenol and 2,4-DCP was studied, and the thermodynamic parameters were calculated. The graph of ln (*K*_T_) versus 1/*T* yields an equation in linear form. From these equations, the parameters such as Δ*H*° and Δ*S*° can be determined as below,

10 $${\Delta H}^{{\text{o}}}=\mathrm{-Slope*R }$$

11 $${\Delta S}^{{\text{o}}}=\mathrm{Intercept*R }$$

where *R* is 8.314 J/mol K, universal gas constant. The plot of ln (*K*_T_) vs 1/*T* depicting the effect of temperature on phenol and 2,4-DCP adsorption is illustrated in Fig. 4. The observed trend indicates a fall in adsorption of phenol and 2,4-DCP, implying an exothermic process. The thermodynamic parameters related to optimum concentration are listed below in Table 3. The negative values of Δ*G*° ranging between 0.673 and 3.653 kJ/mol depict adsorption process to be feasible and spontaneous (details listed in supplementary data Table S7). Further, an increase in Δ*G*° values with rise in temperature from 283 to 323 K indicates descend in adsorption. The negative values of Δ*H*° = 22 kJ/mol depict exothermic adsorption and less than 40 kJ/mol shows that phenol and 2,4-DCP adsorption on CFPAC was dominated by physical adsorption (Sriramoju et al. 2021). The negative entropy change, i.e. Δ*S*° = 0.066, signifies decline in the randomness at the interface of CFPAC and the chosen adsorbate (phenol and 2,4-DCP) solution (Wang et al. 2023). The obtained values were consistent with study carried out by Badu and Mojoudi (Badu Latip et al. 2021; Mojoudi et al. 2019). The data of all the thermodynamic parameters (Δ*H*°, Δ*S*° and Δ*G*°) are available in the supplementary data Table S7.

**Table 3:** Thermodynamic parameters for adsorption of Phenol and 2,4-DCP on CFPAC

Pollutant	*C*_o_ (mg/L)	∆*G* (kJ/mol)					∆*H* (kJ/mol)	∆*S* (kJ/mol K)
		283 K	293 K	303 K	313 K	323 K		
Phenol	25	− 3.395	− 3.653	− 1.117	− 0.673	− 1.685	− 22.161	− 0.066
2,4-DCP	50	− 7.060	− 6.489	− 5.918	− 5.347	− 4.776	− 23.22	− 0.057

#### Isosteric heat of adsorption

Another parameter called as isosteric heat of adsorption (Δ*H*_x_ kJ/mol) was also investigated to identify the type of mechanism that governs the adsorption process. It holds a vital importance in the context of adsorption plant and process design. A plot of ln (*C*_e_) vs 1/*T* as shown in Fig. S9 is obtained. Each set of points are linear fit, and slope of the plot was used to arrive at Δ*H*_x_ (kJ/mol). For an adsorption system, if Δ*H*_x_ is less than 80 kJ mol^−1^, it is said to be dominated by a physical type of adsorption. A system would be dominated by chemical adsorption if, Δ*H*_x_ ranges between 80 and 400 kJ/mol. The data of the present investigation, for phenol adsorption, Δ*H*_x_, was found to vary between 0.59 and 15.95 kJ/mol. Further, for 2,4-DCP, the value varied between 3.12 and 24.14 kJ/mol. Thus, it can be concluded that adsorption of phenol and 2,4-DCP is dominated by physical adsorption and in addition validating the thermodynamic findings. The deviation of Δ*H*_x_ with constant surface loading (mg/g) is illustrated in Fig. S9(c) and (d). Similar plots were also documented for removal of phenol using Mahua seed carbon (Singh et al. 2016). It can be deduced that, there was a drop in the value of Δ*H*_x_ with increase in surface loading (*q*_e_). The information regarding the same are reported in supplementary data Table S8. This further indicated that, the surface of CFPAC to be energetically heterogeneous. The exponential dip in the Δ*H*_x_ with *q*_e_ can be attributed to interaction between pollutant and adsorbent proceeded by adsorbate–adsorbate interaction. At low surface loading values say 10 mg/g and 40 mg/g as observed from Fig. S9(c) and (d), adsorbate-adsorbent interaction is dominant resulting in high Δ*H*_x_. As the surface loading increases, the dominance shifts towards adsorbate–adsorbate interaction (Iheanacho et al. 2023). Comparing the heat of adsorption of both the pollutants, it can be concluded that Δ*H*_x(2,4-DCP)_ was greater than ΔH_x(phenol)_, thus indicating higher interaction between 2,4-DCP and CFPAC.

### Plausible mechanism of adsorption of phenol and 2,4-DCP

The possible mechanisms that could be observed in the adsorption process of phenol and 2,4-DCP are governed by chemisorption (pore diffusion, electrostatic interaction, hydrogen bonding and π-π interactions) with little participation of physical adsorption (Bhatnagar and Minocha 2009).

**Fig. 5:**
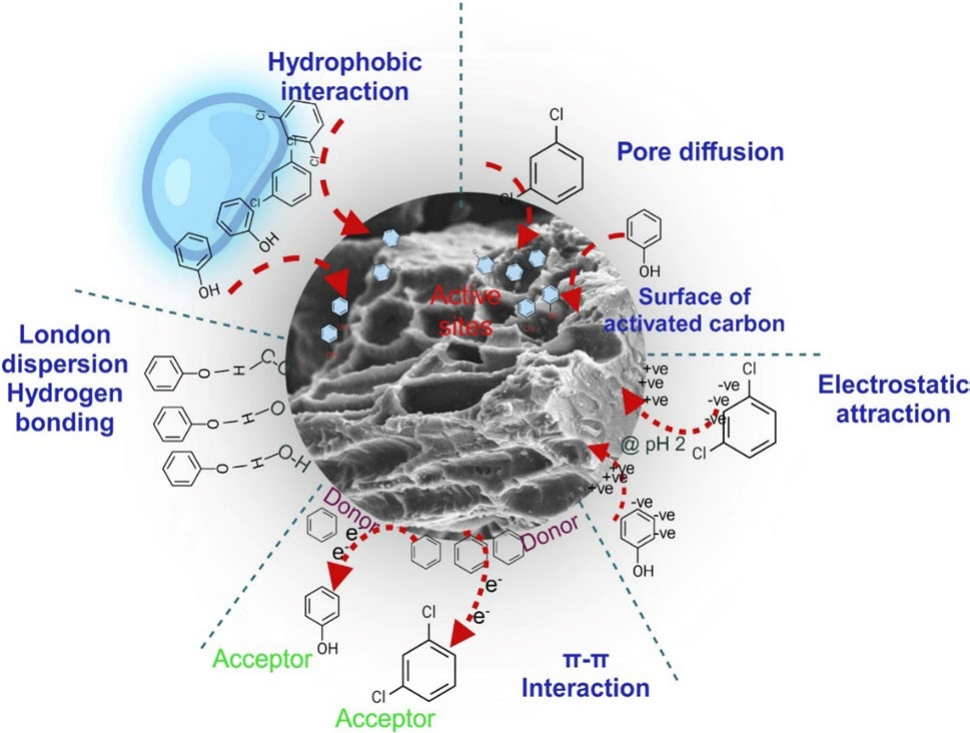
Summary of different mechanism in adsorption of phenol/2,4-DCP onto CFPAC

From the IPD model, it can be interpreted that, there is an occurrence of intra-particle diffusion during the adsorption of phenols. Further, this could be confirmed by the regression coefficient as shown in Table 2, that external diffusion had greater contribution. The effect can also be supported by the change in pore size which can be observed in SEM-EDS images in Fig. S10(a) and (b). From the effect of pH on adsorption as represented in Fig. 2e, it could be concluded that acidic pH was favouring adsorption process (Pei al. 2023b). Thus, resulting in electrostatic attraction, where phenol/2,4-DCP was attracted towards positively charged adsorbent. As corroborated by FTIR peaks of before and after adsorption in Fig. S11 (detail shown in supplementary data), it is possible that weak hydrogen bonding also played a role in adsorption of both pollutant (to a lesser extent). This bonding takes places when water molecules get adsorbed to surface oxygen group (Zhang et al. 2023). Hydrogen bonding could be due to interaction of oxygen, hydrogen groups of adsorbents with alkyl hydrogen of adsorbate (Iheanacho et al. 2023). The FTIR spectra verifies presence of O–H, C–H and C–O. Further, phenol and 2,4-DCP are benzene link aromatic compounds. A donor–acceptor link could be established due to these benzene rings. Thus, stacking of phenol and 2,4-DCP onto adsorbent surface results in π- π interaction where phenolic compound acts as acceptor and acidic surface acts as donor (Kumar et al. 2022). Hydrophobic interactions are considered to be contributing towards adsorption of phenols thereby addressing increased adsorption in the study. A summary of all the mechanism is also shown in the Fig. 5

### Spiked studies

**Fig. 6:**
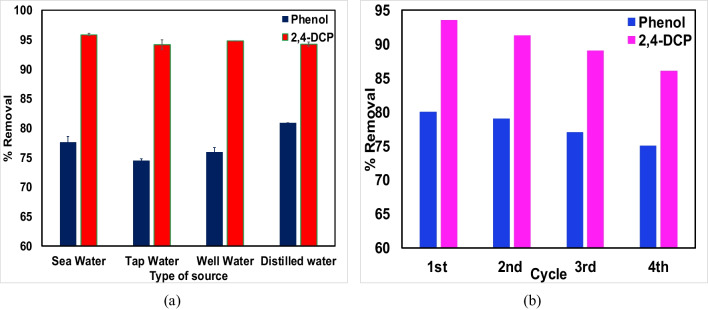
** a** Spiked studies in different water samples and **b** desorption/regeneration

These investigations are carried to understand the possible feasibility of removal of pollutants from natural water samples. The graph of spiked studies of phenol and 2,4-DCP in various water samples is shown Fig. 6a. It displays higher %removal (> 75%) for phenol and (> 93%) for 2,4-DCP. Further, it can also be noticed that removal of phenol and 2,4-DCP was higher in spiked seawater. This could be due to salinity of water which is supporting in additional removal of pollutants.

### Desorption/regeneration studies

Reusability of the spent carbon has a substantial potential in terms of economic benefit. The investigations reported in the literature (Song et al. 2023; Supong et al. 2020) have suggested the likelihood. In this study, it was observed that, the mass of adsorbent after four consecutive cycles of adsorption–desorption was reduced. The loss was approximately 38–40% of initial material used. From Fig. 6b, it can be observed that removal efficiency for phenol and 2,4-DCP declined to 74% and 86% after 4 cycles. This was due to unavailability of active sites (Kumar et al. 2022). Meanwhile, the desorption efficiency dropped approximately to 90%. The initial higher % of desorption was due to the fact that the solid-phase phenol gets converted to sodium phenolate/chlorophenolate when it comes in contact with NaOH solution. Similar results were also reported on desorption of phenol from *Borassus flabellifer* fruit husk–activated carbon (Sathya Priya and Sureshkumar 2020). The investigation thus, demonstrates that the uptake of both the pollutants was reversible.

### Cost analysis

The cost for the production of 1 kg of activated derived from *Cassia fistula* was quantified for lab scale. The total cost includes expenditure on electricity usage, chemicals and water. The cost of procurement of pods was not included as the source was available near to campus. It was found that the chemicals (orthophosphoric acid and sodium bicarbonate) and electricity were major and minor contributor to total cost. Further, the cost of distilled water was least. However, the price of water can be reduced by using normal water. The total cost of production as obtained was $5.46 or 452.5 INR which was considerably less from the commercial activated carbon (Vukelic et al. 2018). The detailed calculation of cost analysis is provided in the supplementary data Table S9.

## Conclusions

To summarise, this study utilised *C. fistula* pods to produce mesoporous activated carbon (CFPAC) at a reasonably lower temperature for activation (500 °C). The resulting material displayed a substantially amorphous, porous structure with an impressive surface area of 1146 m^2^/g. Moreover, the elemental composition of the developed adsorbent revealed higher carbon percentage (90.65%). Notably, the adsorbent exhibited remarkable removal efficiency of 80% for phenol and 93% for 2,4-DCP, accompanied by significant adsorption capacities of 183.79 mg/g and 374.4 mg/g for phenol and 2,4-DCP  respectively at pH 2. Additionally, isotherm data analysis revealed a better fit to the RP model for both pollutants with *R*^2^ value > 0.9740. The kinetic studies emphasised the dominance of pseudo-second-order kinetic, indicating that chemisorption plays a significant role in the adsorption of both pollutants. Thermodynamic investigations depicted negative enthalpy change (Δ*H*°) with magnitude less than 40 kJ/mol, suggesting physical adsorption favoured the process. Further, the observed lower activation energy (*E*_a_) between 5 and 40 kJ/mol provided additional insight, indicating the prevalence of physisorption. The values obtained from isosteric heat of adsorption of phenol and 2,4-DCP were also found to be consistent with thermodynamic data. Additionally, the prepared adsorbent demonstrated significant potential in treating phenolic pollutants in spiked water samples, achieving noteworthy removal of phenolic pollutant (> 90%). Further, desorption studies reveal that the process of adsorption in reversible for both the pollutants. These findings underscore the viability of *C. fistula* pods as a promising and cost-effective natural resource to produce surface-active carbon, offering an environmentally friendly solution for effective pollutant removal.

## Supplementary Information

Below is the link to the electronic supplementary material.

Supplementary file1 (DOCX 2322 KB)

## Data Availability

The data from the current study are available from the corresponding author upon reasonable request.
