# Supplementary material for: Enhanced adsorption of phenolic compounds using biomass-derived high surface area activated carbon: Isotherms, kinetics and thermodynamics
Source: Environ Sci Pollut Res Int. 2024 Apr 5;31(60):67442–60. doi: 10.1007/s11356-024-32971-1 (PMC11685270; doi:10.1007/s11356-024-32971-1)
Supplement: Supplementary file 1 — Supplementary file1 (DOCX 2322 KB) [file 11356_2024_32971_MOESM1_ESM.docx]

**Enhanced adsorption of phenolic compounds using biomass-derived high surface area activated carbon: Isotherms, Kinetics and Thermodynamics**

Praveengouda Patil^1^, Gautham Jeppu^1^, Manjunath Singanodi Vallabha^2^, Chikmagalur Raju Girish^1*^

^1^Department of Chemical Engineering, Manipal Institute of Technology,

Manipal Academy of Higher Education, Manipal-576104, Karnataka, India

^2^Department of Civil Engineering, BMS College of Engineering, Bengaluru-560019, India

*Corresponding author email id: [cr.girish@manipal.edu](mailto:cr.girish@manipal.edu)

# SUPPLEMENTARY FILE

**List of Tables**

# Table S1. Impacts of phenolic compounds on human being.

# Table S2. List of Isotherms used in the present investigation.

# Table S3. Kinetic models: linear and non-linear forms.

# Table S4. Proximate and Ultimate analysis.

# Table S5. Elemental composition before and after adsorption from EDS.

# Table S6. Optimum operational conditions for isotherm studies.

# Table S7. Thermodynamic parameters for adsorption of Phenol and 2,4-DCP on CFPAC.

# Table S8. Isosteric heat of adsorption parameters for phenol and 2,4-DCP.

# Table S9. Cost analysis for preparation of CFPAC.

# Table S1. Impacts of phenolic compounds on human being.

| Exposure | Hazards |
| --- | --- |
| Inhalation | Irritation in respiratory system |
| Oral | Fatal death |
| Eyes | Severe damage and blindness |
| Skin | Irritation and corrosive damage to skin, burns are developed on skin |
| Chronic exposure | Headache, nausea, issue in swallowing, vomit, and diarrhoea. The phenol can also damage liver and kidney |

# Table S2. List of Isotherms used in the present investigation

| Isotherm | Equation | | Ref. |
| --- | --- | --- | --- |
|  | Linear Form | Non-Linear form |  |
| Langmuir Isotherm | $\frac{C_{e}}{q_{m}}=\frac{1}{K_{L}*q_{m}}+\frac{C_{e}}{q_{m}}$ | $q_{e}=\frac{q_{m}*K_{L}*C_{e}}{1+K_{L}*C_{e}}$ | (Girish and Ramachandra Murty 2014) |
| Freundlich Isotherm | $\mathrm{lnq}_{e}=\frac{1}{n}\mathrm{lnC}_{e}+\mathrm{lnK}_{F}$ | $q_{e}=K_{F}*C_{e}^{\left( \frac{1}{n} \right)}$ | (Karri, Sahu, and Jayakumar 2017) |
| Redlich-Peterson model | $\ln\left( \frac{C_{e}}{q_{e}} \right)=\beta\mathrm{lnC}_{e}-\mathrm{lnA}_{\mathrm{RP}}$ | $q_{e}=\frac{A_{\mathrm{RP}}*C_{e}}{1+K_{\mathrm{RP}}*C_{e}^{\beta}}$ | (Dehmani et al. 2022) |
| Temkin model | $q_{e}=\frac{\mathrm{RT}}{b_{T}}*ln\left( A_{T} \right)+\frac{\mathrm{RT}}{b_{T}}*ln\left( C_{e} \right)$ | $q_{e}=\frac{\mathrm{RT}}{b_{T}}*ln\left( A_{T}*C_{e} \right)$ | (Gama et al. 2022) |

where, q_m_ is maximum quantity of phenol/2,4-DCP on unit mass of CFPAC, C_e_ is aqueous concentration of pollutant (phenol/2,4-DCP) at equilibrium, K_L_, K_F,_ A_RP,_ K_RP_, $\beta$, A_T_, b_T_ are constants of Langmuir, Freundlich, RP isotherm and Temkin isotherm.

# Table S3. Kinetic models: linear and non-linear forms

| **Types of equation** | **Linear form** | **Non-linear form** | **References** |
| --- | --- | --- | --- |
| Pseudo-first-order | $\ln\left( q_{e}-q_{t} \right)=ln\left( q_{e} \right)-k_{1}*t$ | $q_{t}=q_{e}*\left( 1-e^{-k_{1}t} \right)$ | (Sriramoju, Dash, and Majumdar 2021) |
| Pseudo-second-order | $\frac{t}{q}=\frac{1}{k_{2}*q_{e}^{2}}+\frac{1}{q_{e}}t$ | $q_{t}=\left( \frac{k_{2}*q_{e}^{2}*t}{1+k_{2}*q_{e}*t} \right)$ | (Mohammadi, Darijani, and Karimi 2020) |
| Intraparticle diffusion | $q_{t}=K_{\mathrm{pi}}*t^{0.5}+C_{i}$ | - | (Lütke et al. 2019) |

Where, q_t_ is adsorption capacity (mg/g) of pollutant at a given time “t”, q_e_ is experimental adsorption capacity of pollutant at equilibrium, k_1_ (min^-1^) and k_2_ (g/mg/min) is rate constant of pseudo-first-order and pseudo-second-order model respectively (Yu et al. 2021). Further, K_pi_ is diffusion constant (mg/g/min^1/2^) and C_i_ is thickness of boundary layer (Kuśmierek, Szala, and Światkowski 2016).

# Table S4. Proximate and Ultimate analysis

| Proximate Analysis | % Moisture | %Volatile matter | % Ash | %Fixed Carbon |
| --- | --- | --- | --- | --- |
| Value (%) | 8.495 | 32.995 | 3.385 | 55.125 |
| Ultimate Analysis | Carbon | Hydrogen | Nitrogen | Sulphur |
| Value (%) | 49 | 7.41 | 0.86 | 0.07 |

# Table S5. Elemental composition before and after adsorption from EDS

| **Condition** | **Element** | **C** | **O** | **Na** | **P** | **K** | **Cl** |
| --- | --- | --- | --- | --- | --- | --- | --- |
| Before adsorption | wt/% | 90.65 | 7.735 | 0.28 | 1.19 | 0.145 | 0 |
| After adsorption: Phenol | wt/% | 90.03 | 8.19 | 0.285 | 1.22 | 0.28 | 0 |
| After adsorption :2,4-Dichlorophenol | wt/% | 87.28 | 8.5 | 0.21 | 0.785 | 0.17 | 3.04 |

# Table S6. Optimum operational conditions for isotherm studies

| **Conditions** | **Phenol** | **2,4-DCP** |
| --- | --- | --- |
| Concentration (mg/L) | 25 | 50 |
| pH | 2 | 2 |
| Temperature (^o^C) | 20 | 20 |
| Agitation speed (rpm) | 150 | 120 |
| Adsorbent dosage (g/L) | 1.6 | 0.6 |

# Table S7. Thermodynamic parameters for adsorption of Phenol and 2,4-DCP on CFPAC

| **Adsorbate** | **Co (mg/L)** | **∆G kJ/mol** | | | | | **∆H kJ/mol** | **∆S kJ/mol/K** |
| --- | --- | --- | --- | --- | --- | --- | --- | --- |
|  |  | **283K** | **293K** | **303K** | **313K** | **323K** |  |  |
| PHENOL | 25 | -3.395 | -3.653 | -1.117 | -0.673 | -1.685 | -22.161 | -0.066 |
|  | 50 | -2.081 | -2.437 | -0.801 | -0.509 | -0.850 | -14.872 | -0.045 |
|  | 100 | -0.756 | -1.170 | 0.087 | 0.715 | 0.167 | -11.642 | -0.038 |
|  | 150 | -0.084 | -0.596 | 0.883 | 1.096 | 0.896 | -10.721 | -0.037 |
|  | 200 | 0.180 | -0.297 | 1.011 | 1.487 | 1.316 | -11.565 | -0.041 |
|  | 300 | 0.819 | 0.209 | 1.624 | 1.726 | 1.014 | -4.967 | -0.020 |
|  | 400 | 1.067 | 0.640 | 2.301 | 2.267 | 1.413 | -5.967 | -0.025 |
|  | 500 | 1.386 | 1.232 | 2.426 | 2.782 | 1.177 | -2.335 | -0.014 |
|  | 600 | 1.534 | 1.429 | 2.334 | 2.636 | 3.159 | -11.148 | -0.111 |
| 2,4-DICHLOROPHENOL | 25 | -7.835 | -7.203 | -6.571 | -5.939 | -5.307 | -25.72 | -0.063 |
|  | 50 | -7.060 | -6.489 | -5.918 | -5.347 | -4.776 | -23.22 | -0.057 |
|  | 100 | -4.887 | -4.459 | -4.030 | -3.602 | -3.173 | -17.01 | -0.043 |
|  | 150 | -3.506 | -3.127 | -2.748 | -2.369 | -1.990 | -14.23 | -0.038 |
|  | 200 | -2.518 | -2.257 | -1.995 | -1.734 | -1.472 | -9.92 | -0.026 |
|  | 300 | -1.239 | -0.937 | -0.636 | -0.335 | -0.034 | -9.76 | -0.030 |
|  | 400 | -0.551 | -0.143 | 0.265 | 0.673 | 1.081 | -12.09 | -0.041 |
|  | 500 | 0.261 | 0.513 | 0.765 | 1.018 | 1.270 | -6.88 | -0.025 |
|  | 600 | 0.247 | 0.715 | 1.183 | 1.652 | 2.120 | -13.01 | -0.047 |

# Table S8. Isosteric heat of adsorption parameters for phenol and 2,4-DCP

| **Pollutant** | **q_e_ (mg/g)** | **ΔH_x_ (kJ/mol)** |
| --- | --- | --- |
| **Phenol** | 10 | 15.953 |
|  | 20 | 9.576 |
|  | 30 | 6.151 |
|  | 40 | 5.034 |
|  | 50 | 5.045 |
|  | 70 | 2.018 |
|  | 90 | 2.172 |
|  | 100 | 0.596 |
| **2,4-DICHLOROPHENOL** | 40 | 24.146 |
|  | 80 | 21.406 |
|  | 140 | 14.257 |
|  | 190 | 10.754 |
|  | 230 | 6.821 |
|  | 280 | 5.626 |
|  | 320 | 5.887 |
|  | 360 | 3.122 |
|  | 390 | 5.210 |

# Table S9. Cost analysis for preparation of CFPAC

| **Description of item** | | **Unit cost** | **No. of Units** | | **Price**  **(INR)** | | **Price**  **(USD)** |
| --- | --- | --- | --- | --- | --- | --- | --- |
| **For 1Kg of CFPAC** | | | | | | | |
| 1. | Biomass | 0 | |  | | 0 | 0 |
| 2. | Electricity | 7.5 | | 4 | | 30 | 0.36 |
| 3. | Chemicals |  | |  | | 380 | 4.59 |
| 4. | DI Water |  | |  | | 24 | 0.29 |
| Total | | | | | | 434 | 5.24 |

# List of Figures

# Fig. S1 Nitrogen adsorption/ desorption isotherms of CFPAC

# **Fig. S2 Non-linear model fit of isotherms for phenol (a), (b) and 2,4-DCP (c), (d) at pH 2 and pH 5**

# Fig. S3 Influence of initial concentration on the separation factor (R_L_)

# Fig. S4 Langmuir isotherm at different temperatures a) Phenol and b) 2,4-DCP

# Fig. S5 Pseudo-First-Order (a) and (c), Pseudo-Second-Order (b) and (d) non-linear kinetics for phenol, 2,4-DCP

# Fig. S6 Pseudo first order and Pseudo second order model fit for Phenol (a) and (b) and 2,4-DCP (c) and (d)

# Fig. S7 Plot of ln(k_2_) vs 1/T for determining activation energy

# Fig. S8 Intraparticle diffusion plot of a) Phenol b) 2,4-DCP

# Fig. S9 Isosteric heat of adsorption for (a) phenol (b) 2,4-DCP on CFPAC and plot of ΔH_x_ vs Loading for phenol (c) and (d) 2,4-DCP

# Fig S10 SEM-EDS after adsorption of phenol and 2,4-DCP

# Fig. S11 FTIR spectra before and after adsorption of phenol and 2,4-DCP

# Fig. S1 Nitrogen adsorption/ desorption isotherms of CFPAC

Fig. S2 Non-linear model fit of isotherms for phenol (a), (b) and 2,4-DCP (c), (d) at pH 2 and pH 5

# Fig. S3 Influence of initial concentration on the separation factor (R_L_)

|  | **** |
| --- | --- |

# Fig. S4 Langmuir isotherm at different temperatures a) Phenol and b) 2,4-DCP

# Fig. S5 Pseudo-First-Order (a) and (c), Pseudo-Second-Order (b) and (d) non-linear kinetics for phenol, 2,4-DCP

# Fig. S6 Pseudo first order and Pseudo second order model fit for Phenol (a) and (b) and 2,4-DCP (c) and (d)

# Fig. S7 Plot of ln(k_2_) vs 1/T for determining activation energy

| 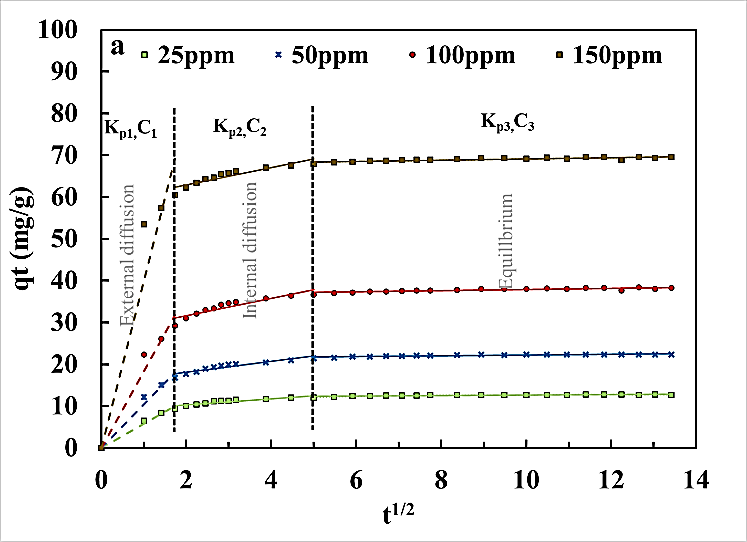 |
| --- |
| 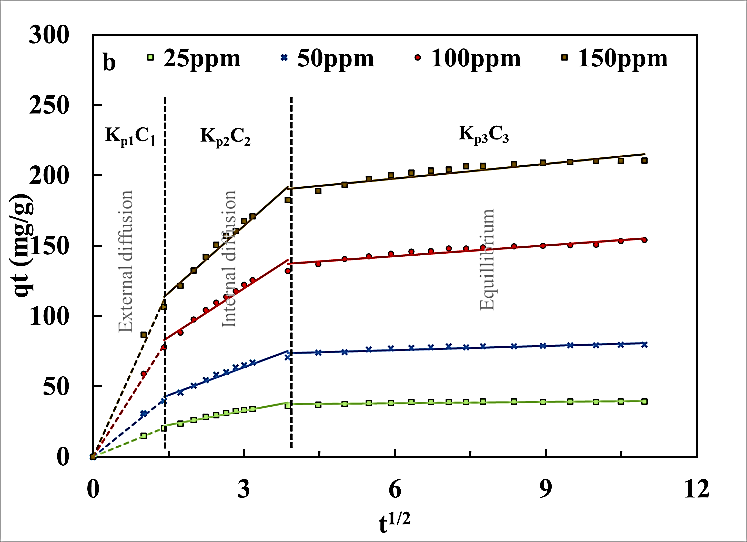 |

# Fig. S8 Intraparticle diffusion plot of a) Phenol b) 2,4-DCP

# Fig. S9. Isosteric heat of adsorption for (a) phenol (b) 2,4-DCP on CFPAC and plot of ΔH_x_ vs Loading for phenol (c) and (d) 2,4-DCP

1. (b)

# Fig S10 SEM-EDS after adsorption of (a) phenol and (b) 2,4-DCP


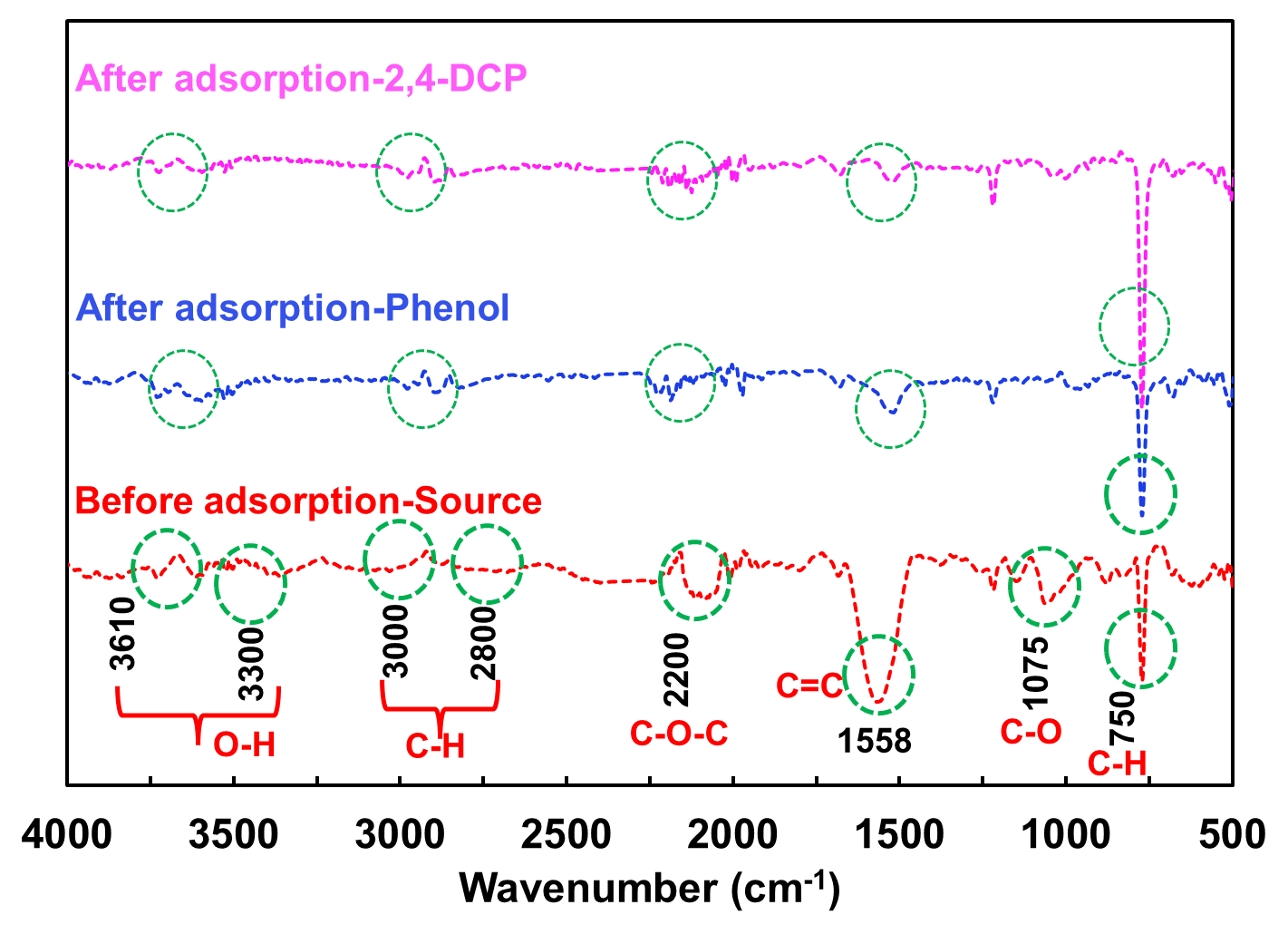


# Fig. S11 FTIR spectra before and after adsorption of phenol and 2,4-DCP
